# Heterophoria in multiple sclerosis patients: a proof of principle cross-sectional study

**DOI:** 10.3389/fimmu.2024.1431394

**Published:** 2024-08-19

**Authors:** Jonas Graf, Margit Weise, Tanja Guthoff, Carolin Balloff, Marcia Gasis, Heike Link, Sebastian Küchlin, Wolf Lagrèze, Sven G. Meuth, Orhan Aktas, Philipp Albrecht

**Affiliations:** ^1^ Department of Neurology, Medical Faculty and University Hospital Düsseldorf, Heinrich-Heine-University Düsseldorf, Düsseldorf, Germany; ^2^ Department of Ophthalmology, Medical Faculty and University Hospital Düsseldorf, Heinrich-Heine-University Düsseldorf, Düsseldorf, Germany; ^3^ Department of Neurology, Maria Hilf Clinics, Mönchengladbach, Germany; ^4^ Department of Ophthalmology, University Hospital, Medical Faculty, Albert-Ludwigs-University Freiburg, Freiburg, Germany

**Keywords:** multiple sclerosis, heterophoria, clinical assessment, screening, orthoptic assessment

## Abstract

**Objectives:**

The pathophysiology of multiple sclerosis (MS) involves inflammatory neurodegeneration in the brainstem, cerebellum, and retina. The clinical relevance of oculomotor involvement in MS, however, remains uncertain.

**Methods:**

In this cross-sectional study, we evaluated heterophoria as a (sub)clinical tool in 54 MS patients and 55 age-matched healthy controls (HCs). We quantified heterophoria in prism diopters for distance and near range with orthoptic examination. Our primary outcome was high degrees of horizontal heterophoria (HDHH) defined as measurements beyond ±2 standard deviations from the mean prism diopter of heterophoria of our HCs.

**Results:**

More than one-third (37%, n=20/54) of MS patients but only 11% (n=6/55) of HCs were classified as HDHH [distance, MS=9% (n=5/54) versus HC=6% (n=3/55); near, MS=19% (n=10/54) versus HC=5% (n=3/55)]. Our MS patients presented more combined vertical and horizontal deviations at near range [MS 19% (n=10/54) versus for HC 7% (n=4/55)]. We observed the combination of HDHH both at distance and at near testing in 9% (n=5/54) of MS patients but not at all in HCs (n=0/55).

**Discussion:**

Despite the high prevalence of heterophoria, HDHH may be an additional (sub)clinical tool of subclinical involvement in MS. Thus, orthoptic examination may be an additional tool to improve MS diagnostic procedures.

## Introduction

The pathophysiology of multiple sclerosis (MS) involves inflammatory neurodegeneration in the brainstem and cerebellum. In-depth neuro-ophthalmological examinations are increasingly relevant for characterizing MS patients (1, 2). While retinal changes in MS have been extensively characterized and associated with disease progression (3, 4), the precise profile and extent of oculomotor involvement in MS compared to healthy control (HCs) remains less certain. Subtle inflammation and/or degeneration of the central nervous system may lead to heterophoria (5) or to manifest heterotropia (6) in MS patients. Considering that heterophoria and/or subclinical binocular vision anomalies in the general population may be considered common (7–9), we hypothesized that heterophoria is also frequently found in MS patients potentially in a more pronounced form. Brainstem/cerebellar pathology is known to be associated with a poor MS prognosis (10) and (subclinical) changes of binocular vision (11, 12). Measurement of heterophoria may become a valuable tool for characterizing MS patients.

## Patients and methods

Patients and HCs were prospectively recruited at the Department of Neurology, Heinrich Heine University, Düsseldorf, Germany between 2018 and 2021. Inclusion criteria were >18 years of age and an MS diagnosis according to the McDonald criteria 2017. A complete and signed declaration of consent was required for participation. Patients with an acute relapse involving vision or brainstem functions and those with manifest oculomotor symptoms or heterotropia were excluded. However, due to the monocentric study design and heterogeneous follow-up times of the patients, we were not able to exclude patients with a history of prior relapses involving the brainstem or optic nerves.

All participants underwent orthoptic examinations following a defined protocol performed by a trained optometrist. Assessments included the alternating cover and Maddox rod test in primary position of the eyes, and full/low (100%/2.5%) habitually corrected contrast visual acuity testing using SLOAN charts of the Early Treatment Diabetic Retinopathy Study (ETDRS, Precision Vision, Inc.) at a distance of 2 m. A unilateral and alternating cover test was performed at 6 m (distance) and 40 cm (near) to separate heterotropia from heterophoria. Horizontal (esophoria/exophoria) and/or vertical (hypophoria/hyperphoria) heterophoria was measured. The Maddox rod and corresponding tangent scale (Maddox cross) were used to quantify the horizontal and vertical deviation in prism diopters for each eye using a prism bar at distance (6 m) and near (40 cm) range. Maddox rod test–retest reliability was assessed with the same structured procedure in an independent cohort of 15 MS patients (**Supplementary Figure S1**) prior to formal study recruitment.

A high degree of horizontal heterophoria (HDHH) was defined as a heterophoria exceeding the mean value ±2 standard deviations of the prism diopter angle in HCs. (**Supplementary Table S1**).

### Statistical evaluations

Statistical analyses were performed using IBM SPSS Statistics (version 20). The intraclass correlation coefficient was calculated using a two-way mixed model and absolute agreement type to evaluate the test–retest reliability of our assessments (**Supplementary Figure S1**).

Group comparisons between patients with MS and HC (independent variable) for continuous data [prism diopter (dependent variable)] were performed using Generalized Estimating Equation (GEE) models [model: constant term, analysis-type III (Wald)] with an exchangeable correlation matrix including all eyes, correcting for within subject inter-eye correlations, age, and sex for strength of deviation (horizontal, vertical) at distance and near range (**Supplementary Table S2**). Probability values (p, two-tailed) <0.05 were considered significant.

To assess and quantify the strength of association between determined heterophorias (i.e., the prism diopter angle measured using Maddox rod test and alternating cover test, distance and near range), HDHH and history of double vision (DV) and group (MS vs. HCs), odds ratio (OR), and the associated 95% confidence interval (95%CI) were calculated. 95%CIs not including 1 were considered statistically significant.

## Results

To investigate reproducibility, we repeated Maddox rod and alternating cover test assessments on consecutive days and at morning and evening in 15 MS patients. Test–retest reliability was excellent with intraclass correlation coefficients >0.85 for all testing conditions (**Supplementary Figure S1**).

**Figure 1:**
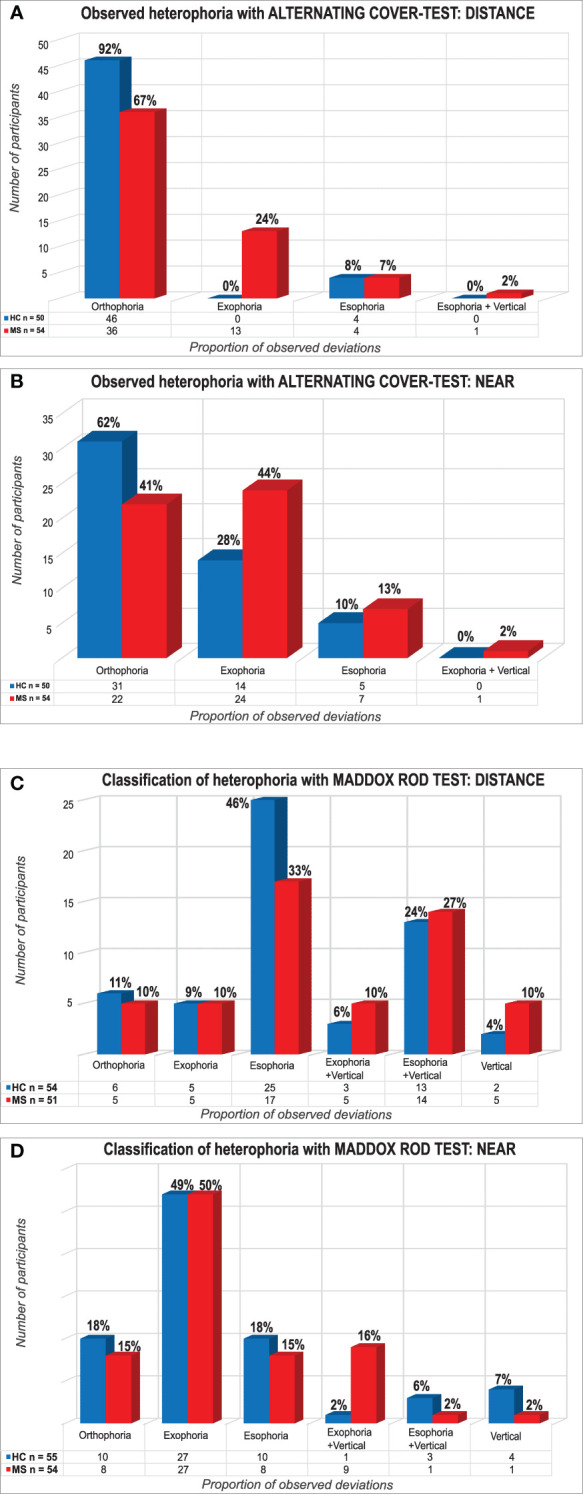
Proportion of multiple sclerosis (MS) patients and healthy controls (HCs) with and without heterophoria. Plots show results of the alternating cover test for distance **(A)** and near **(B)** range and the Maddox rod method for distance **(C)** and near **(D)** range, grouped by orthophoria, exophoria, and esophoria (“+ vertical” indicates the presence of an additional vertical deviation). Lack of Measurement data for five HCs **(A, B)**. One HC and three MS patients were excluded because of suppression of fixation light distance **(C)**.

We included a cohort of 54 MS patients with a predominantly relapsing disease course (67% of patients) and 55 age-matched HCs (**Table 1** and **Supplementary Figure S2**). The mean age of MS patients was 41.9 ± 11.8 years. The mean disease duration was 11.9 ± 9.3 years, with a mean EDSS of 3.4 ± 2.2. The alternating cover test revealed that a higher proportion of MS patients had exophoria both at distance [MS n=13/54 (24%) versus HCs n=0/50 (0%), OR=1.32 (95%CI: 1.13–1.53)] and in near range [MS n=24/54 (44%) versus HCs n=14/50 (28%), OR=2.06 (95%CI: 0.91–4.67), **Figures 1A, B**] compared to HCs. Upon Maddox rod testing, a more complex picture emerged with higher proportions of heterophoria and combined deviations in both MS patients and HCs [e.g., exophoria near; MS n=27/54 (50%) versus HCs n=27/55 (49%), **Figures 1C, D**]. To scrutinize if unidirectional or combined heterophoria forms have a greater potential for distinguishing MS patients and HCs, we characterized heterophoria patterns (**Supplementary Figures S3A, B**). Here, our MS patients presented a trend to more combined horizontal/vertical deviations than HCs at near [MS n=10/54 (19%) versus HCs n=4/55 (7%), OR=2.90 (95%CI: 0.85–9.89)] and at distance [MS n=19/51 (37%) versus HCs n=16/54 (30%), OR=1.41 (95%CI: 0.63–3.18)]. Considered individually, the presence of horizontal and vertical deviations was similar between both groups (**Supplementary Table S2**). The degree of horizontal phorias was more broadly distributed in MS patients compared to HCs, while the distribution of the degree of vertical phorias was similar between groups at both distances (**Supplementary Figures S3A, B**). The mean prism diopter value of vertical phorias did not deviate from zero prism diopters [(mean prism diopters ± SD) distance: MS 0 ± 0.7 prism diopters versus HCs 0 ± 0.5 prism diopters, p=0.689; near: MS 0 ± 0.7 prism diopters versus HCs 0 ± 0.5 prism diopters, p=0.881, **Supplementary Figure S4**]. Therefore, we focused on horizontal heterophoria analyses. The prevalence of horizontal heterophoria was similar between groups [**Supplementary Table S2**, e.g., esophoria distance: MS n=17/51 (33%) versus HCs n=25/54 (46%), OR=0.58 (95%CI: 0.26–1.28)]. The mean prism diopter value for the horizontal axis was shifted towards esophoria at distance and towards exophoria at near examination, in both HCs and MS cohort. The mean prism diopter value for exophoria was significantly more pronounced at near examination in MS patients [(mean prism diopters ± SD) distance: MS +0.8 ± 3.7 prism diopters versus HCs +1.4 ± 2.2 prism diopters, p=0.299, GEE; near: MS −4.5 ± 6.3 prism diopters versus HCs −2.3 ± 3.9 prism diopters, p=0.014, GEE]. We observed marked horizontal heterophoria in MS patients: 37% of MS patients (n=20/54) classified as HDHH, as compared to 11% of HCs [n=6/55; distance: OR=1.85 (95%CI: 0.42–8.17); near: OR=3.94 (95%CI: 1.02–15.22), **Supplementary Figure S5**, **Supplementary Table S2**]. We observed the combination of HDHH both at distance and at near testing in n=5/54 (9%) MS patients but not at all in HCs, OR=1.1 (95%CI: 1.01–1.2). This was particularly prominent for exophoric deviations at near range: we found a significantly larger proportion of HDHH in our MS cohort, associated with higher angles of deviation. In order to take into account the influence of other clinical parameters such as previous optic neuritis (ON) or history of previous double vision (DV)/diplopia, these parameters (**Table 1**) were also determined. According to records and history, 27 (50%) of our 54 MS patients had previous ON [MS-HDHH n=10/20 (50%), MS-non-HDHH n=17/34 (50%)]. However, the proportion of patients with a history of DV at any time point after MS diagnosis was significantly larger in the HDHH group with a moderate association [MS-HDHH n=16/20 (80%), MS-non-HDHH n=14/34 (41%), OR=5.71 (95%CI: 1.57–20.78)].

**Table 1:** Demographic, clinical, and visual characteristics of the multiple sclerosis (MS) patients and healthy control (HC) participants.

Characteristics	MS patients			HC participants		
	TOTAL n = 54	HDHH n = 20 (37%)	Non-HDHH n = 34 (63%)	Total n = 55	HDHH n = 6 (11%)	Non-HDHH n = 49 (89%)
*Demographic*						
**Age in years; mean (SD)**	41.9 (± 11.8)	39.8 (± 13.4)	43.2 (± 10.9)	41.5 (± 13.2)	49.3 (± 8.7)	40.6 (± 13.4)
**Female, n (%)**	39 (72)	12 (60)	27 (79)	35 (64)	2 (33)	33 (67)
*Visual*						
**HCVA; LogMAR** **mean (SD)**	0.07 (± 0.32)	0.09 (± 0.23)	0.05 (± 0.36)	-0.04 (± 0.1)	-0.01 (± 0.1)	-0.04 (± 0.1)
**LCVA (2.5%); Letter** **mean (SD)**	25 (± 14)	21 (± 14)	27 (± 13)	40 (± 8)	38 (± 9)	40 (± 8)
*Clinical*						
**RRMS, n (%)**	36 (67)	11 (55)	25 (73)	N/A	N/A	N/A
**SPMS, n (%)**	13 (24)	7 (35)	6 (18)	N/A	N/A	N/A
**PPMS, n (%)**	5 (9)	2 (10)	3 (9)	N/A	N/A	N/A
**Age at symptom onset, year; mean (SD)**	29.5 (± 9.2)	26.1 (± 8.6)	32.1 (± 9.4)	N/A	N/A	N/A
**Age at diagnosis,** **year; mean (SD)**	32.5 (± 10.6)	28.6 (± 11.3)	34.8 (± 9.7)	N/A	N/A	N/A
**Disease duration in year; mean (SD)**	11.9 (± 9.3)	12.5 (± 9.2)	11.4 (± 9.4)	N/A	N/A	N/A
**EDSS at orthoptic assessment, mean (SD)**	3.4 (± 2.2)	4.2 (± 2.3)	3.0 (± 2.1)	N/A	N/A	N/A
**History of ON pat., n (%)**	27 (50)	10 (50)	17 (50)	N/A	N/A	N/A
**History of diplopia, n (%)**	30 (55.6)	16 (80)	14 (41)	N/A	N/A	N/A
*Therapy*						
**None, n (%)**	17 (32)	6 (30)	11 (33)	N/A	N/A	N/A
**Platform****^a^****, n (%)**	12 (23)	3 (15)	9 (27)	N/A	N/A	N/A
**High efficacy****^b^****, n (%)**	24 (45)	11 (55)	13 (40)	N/A	N/A	N/A

EDSS, expanded disability status scale; ON, optic neuritis; SD, standard deviation; H/LCVA, high/low contrast visual acuity; RR/SP/PPMS, relapsing–remitting/secondary progressive/primary progressive multiple sclerosis, HDHH, high degree of horizontal heterophoria (outside the defined limits for horizontal heterophoria angles); non-HDHH, the part of the cohort inside of the defined limits for horizontal heterophoria.

a Platform therapies were defined as Glatirameracetat, Dimethylfumarat, Teriflunomid, Interferon-ß-1b s.c., and Peginterferon.

b High-efficacy therapies were defined as Fingolimod, Ocrelizumab, Natalizumab, Alemtuzumab, Rituximab, Mitoxantron, Cladribin, and Azathioprin.

## Discussion

Characterizing oculomotor MS manifestations has been of special interest since the very first description of the disease by Charcot and the MS-related visual symptoms described by Uhthoff more than a century ago (13), as abnormalities of the afferent visual system and eye movements are common at any stage and course of the disease (14, 15). While these are accepted as integral parts of the clinical picture (1, 16), systematic characterizations of subclinical changes in binocular vision in MS based on orthoptic examination are lacking (17). In our cohort, we observed a higher prevalence of higher degrees of heterophoria in MS patients versus HCs. Specifically, HDHH was found in 37% of MS patients, more than three times the rate observed in HCs (11%). These orthoptic abnormalities may reflect subtle brainstem and cerebellar involvement in disease. These deviations may direct clinicians to investigate infratentorial demyelination, a known prognostic factor (10). To summarize, MS is a disease of the central nervous system, and inflammatory infiltrates involving the brain stem have commonly been identified by MRI imaging and histopathology (18, 19). While these often result in overt clinical manifestations such as oculomotor symptoms like double vision, we suggest that the high rates of HHO observed in our study are likely to be a subclinical manifestation of such pathology involving the oculomotor system in the brainstem. Further studies including high-resolution brain stem MRI imaging are warranted to corroborate this assumption.

Strengths of our study include the use of two independent orthoptic examination techniques, the exclusion of heterotropia, a good test–retest reliability, and the comparison with HCs. Limitations include the rather small sample size and the cross-sectional study design, which precludes prognostic or diagnostic conclusions from our observations. The examiner of the orthoptic measurements was not masked and was therefore aware of the subject’s diagnosis. In addition, no (cycloplegic) refraction or assessment of possible accommodation disorders and the influence of reduced visual acuity compared to the HCs was performed. Likewise, an effect of possible fatigue in the MS cohort cannot be completely ruled out. We cannot exclude that a history of ON may have influenced our findings. However, the similar rates of previous ON in the HDHH and the non-HDHH groups argue against a major influence of a history of ON.

At the same time, the higher rates of a history of a previous DV in the HDHH group may suggest that the HDHH may result from previous lesions involving the oculomotor system, which have since resolved. Thus, the presence of a higher grade heterophoria might be promising as a (sub)clinical tool for MS lesions that involve the oculomotor system. The relevance of our results should be confirmed in larger, longitudinal studies.

## Data Availability

The original contributions presented in the study are included in the article/**Supplementary Material**. Further inquiries can be directed to the corresponding author.
