# Supplementary material for: Heterophoria in multiple sclerosis patients: a proof of principle cross-sectional study
Source: Front Immunol. 2024 Aug 19;15:1431394. doi: 10.3389/fimmu.2024.1431394 (PMC11366644; doi:10.3389/fimmu.2024.1431394)

<u>Supplement</u>

Supplementary Table 1:
Definition of high degrees of horizontal heterophoria (HDHH).
Heterophoria limit values defined as the mean prism diopter of health controls (HCs) plus (esophoria) or minus (exophoria) two standard deviations of the mean prism diopter. Hence, any Maddox rod examination with a value beyond these heterophoria limit values considered a HDHH. Fixation point distance = 6 m, and fixation point near = 40 cm

	**Horizontal Maddox Rod examination**	**Mean (SD) prism diopter of HCs**	**Heterophoria Limit Values in prism diopter (mean ±2x SD)**	**Number of MS patients meeting the definition, n (%)**	**Number of HCs meeting the definition, n (%)**
**Distance**	Exophoria (-)	1.25 (2.08)	1.25 – (2*2.08) ~ **-3**	1 (20%)	0 (0%)
	Esophoria (+)	1.25 (2.08)	1.25 + (2*2.08) ~ **+5**	4 (80%)	3 (100%)
**Near**	Exophoria (-)	-1.95 (3.37)	-1.95 – (2*3.37) ~ **-9**	9 (90%)	3 (100%)
	Esophoria (+)	-1.95 (3.37)	-1.95 + (2*3.37) ~ **+5**	1 (10%)	0 (0%)
**Distance and Near (combined)**	Exophoria (-)	1.25 (2.08)<br>-1.95 (3.37)	~ -3<br>~ -9	4 (80%)	0 (0%)
	Esophoria (+)	1.25 (2.08)<br>-1.95 (3.37)	~ +5<br>~ +5	1 (20%)	0 (0%)

Supplementary Table 2:

Comparison of horizontal as well as vertical deviations between multiple sclerosis patients and healthy controls. Odds ratios (OR) with 95% confidence interval (95% CI) were calculated using the exchangeable generalized estimating equation (GEE) with probability calculation (P) applied to continuous data (mean prism diopters)

	Deviation	*OR*	*95%CI*		*P*
			Lower	*Upper*	
**Alternating Cover Test**					
Distance	**Exophoria**	**1.32**	**1.13**	**1.53**	**<0.001**
Near	Exophoria	2.06	0.91	4.67	0.082
**Maddox Rod Test**					
Distance	Horizontal	0.61	0.28	1.31	0.203
	Esophoria	0.58	0.26	1.28	0.175
	Exophoria	1.07	0.29	3.92	0.924
	Vertical	2.83	0.52	15.27	0.210
	Combined horizontal-vertical	1.41	0.63	3.18	0.407
Near	Horizontal	0.90	0.41	1.98	0.789
	Esophoria	0.78	0.28	2.16	0.636
	Exophoria	1.04	0.49	2.20	0.924
	Vertical	0.24	0.03	2.23	0.176
	Combined horizontal-vertical	2.90	0.85	9.89	0.079
**HDHH**					
	Distance	1.85	0.42	8.17	0.412
	**Near**	**3.94**	**1.02**	**15.22**	**0.035**
	**Distance and Near (combined)**	**1.10**	**1.01**	**1.20**	**0.021**
**Mean prism diopter**					
Horizontal	Distance				0.299
	**Near**				**0.014**
Vertical	Distance				0.603
	Near				0.918

Key: OR = Odds Ratio as the effect size of the relationship: greater than 1 (e.g. HDHH Near highlighted in bold) indicate that the event is more likely to occur as the predictor increases; 95%CI = Confidence interval with 95% confidence level as well as lower and upper limit; HDHH = High degrees of horizontal heterophoria (Esophoria, Exophoria) with the strongest effect size for near range, although within a wide confidence range; GEE = generalized estimating equation for probability calculation (P) with all eyes corrected for age and gender with significance ($p < 0.05$) of the horizontal deviation at near range.

Supplementary Figure 1:
Maddox rod test-retest reliability assessment of 15 multiple sclerosis (MS) patients to account for possible relevant measurement variability. Maddox rod tests repeated in the morning and evening (A) and on two different days (B) for distance and near. Reliability was calculated using the intraclass correlation coefficient (ICC; distance morning/evening ICC = 0.95; near morning/evening ICC = 0.94; distance 1st day/2nd day ICC = 0.86; near 1st day/2nd day ICC = 0.94). For the alternating cover test (C), the intraclass correlation coefficients were also calculated using two measurement points in the morning and evening and on two different days for horizontal deviation (exophoria, esophoria) at distance and near (distance morning/evening ICC = 0,86; near morning/evening ICC = 0.81; distance 1st day/ 2nd day ICC = 0.68; near 1st day/ 2nd day ICC = 0.87). The ICC values shows also a good strength of agreement.

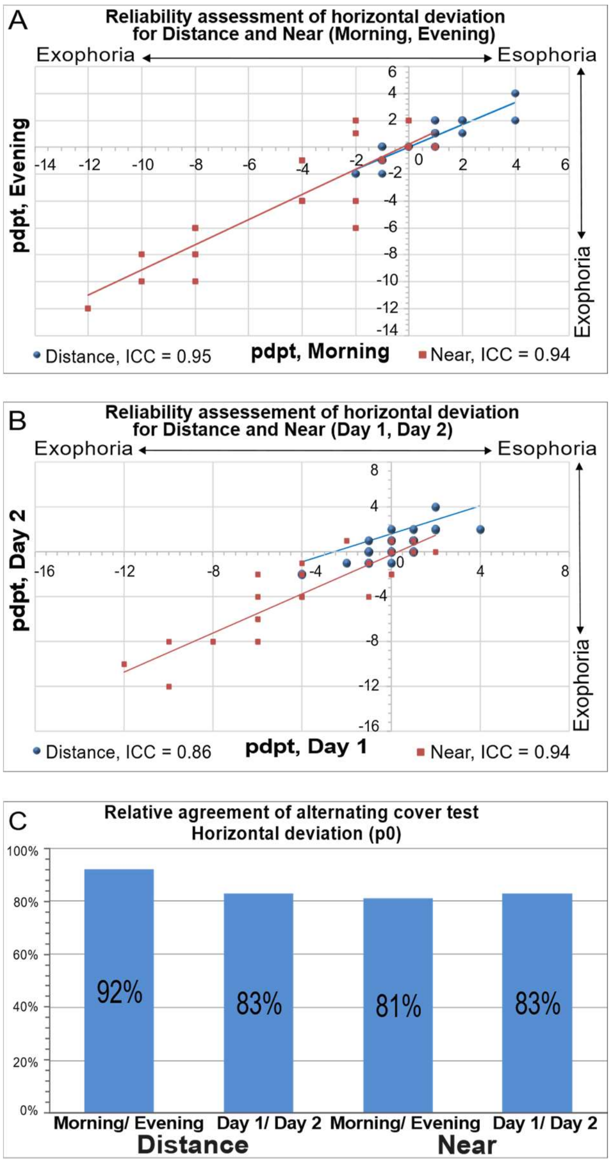

Key: pdpt = prism diopter

Supplementary Figure 2: Recruitment plan

Flowchart of the subject recruitment at the Department of Neurology, Heinrich Heine University Düsseldorf.

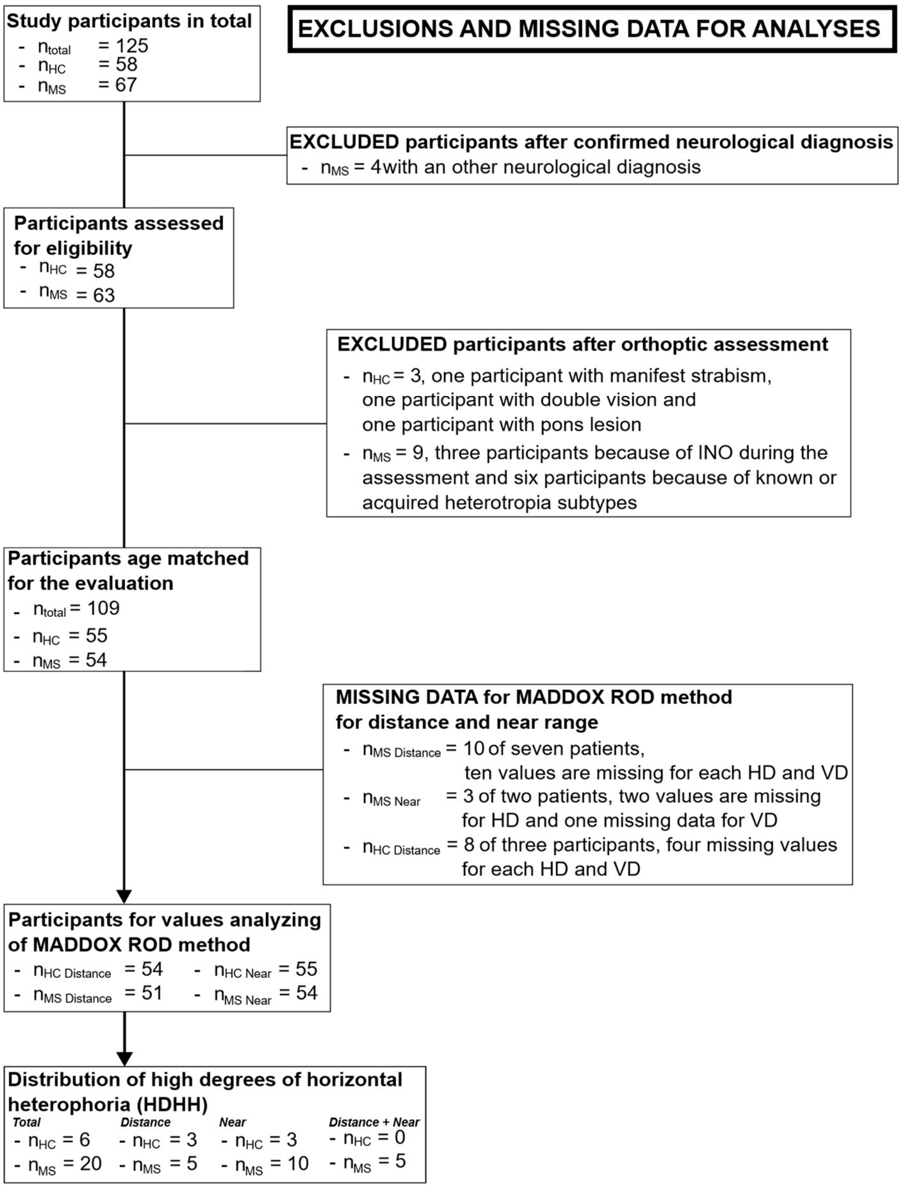

Key: HD = horizontal deviation, VD = vertical deviation

Supplementary Figure 3:

Number of multiple sclerosis (MS) patients and health controls (HCs) with unidirectional (0 prism diopter [pdpt]) and combined deviation [horizontal (exophoria, esophoria), vertical (hyperphoria, hypophoria)] grouped by quantified Maddox rod test at distance (A, C) and near (B, D) and high degree of horizontal heterophoria (HDHH; C and D). 3 MS patients and 1 HC (distance) were excluded due to suppression of fixation light for A and C.

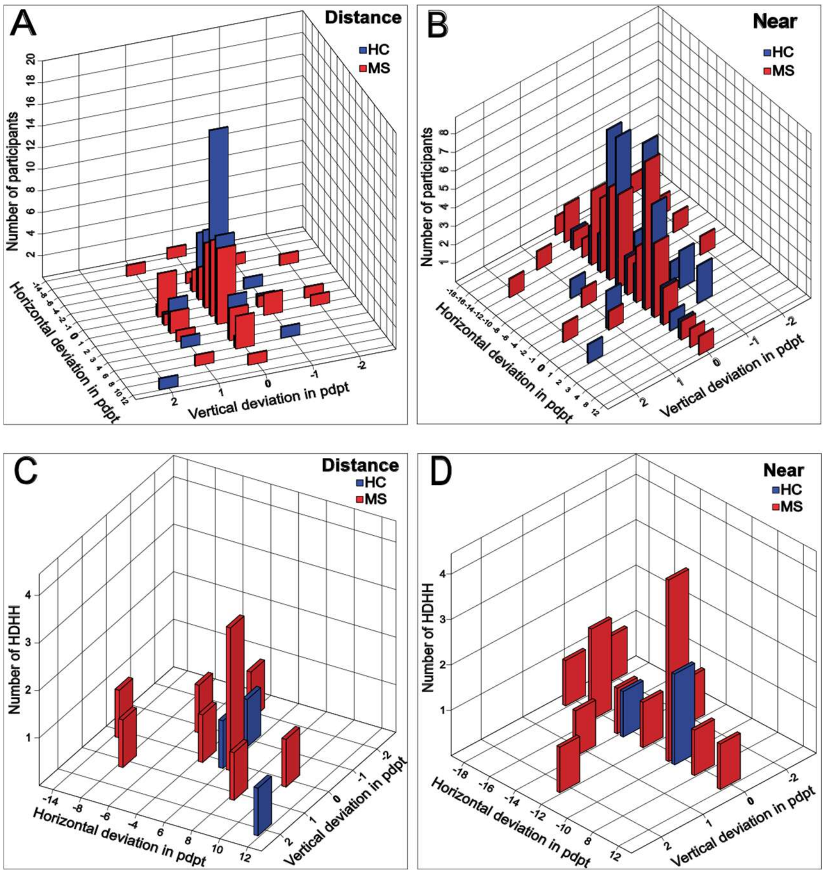

Supplementary Figure 4:

Standard distribution of quantified Maddox rod measurement results in multiple sclerosis (MS) patients and health controls (HCs) in prism diopter grouped by distance (A and B) and near (C and D) examinations. High degrees of horizontal heterophoria (HDHH) in A and C indicated by red vertical line (limit values). 3 MS patients and 1 HC (A) were excluded due to suppression of fixation light for A, B, and D.

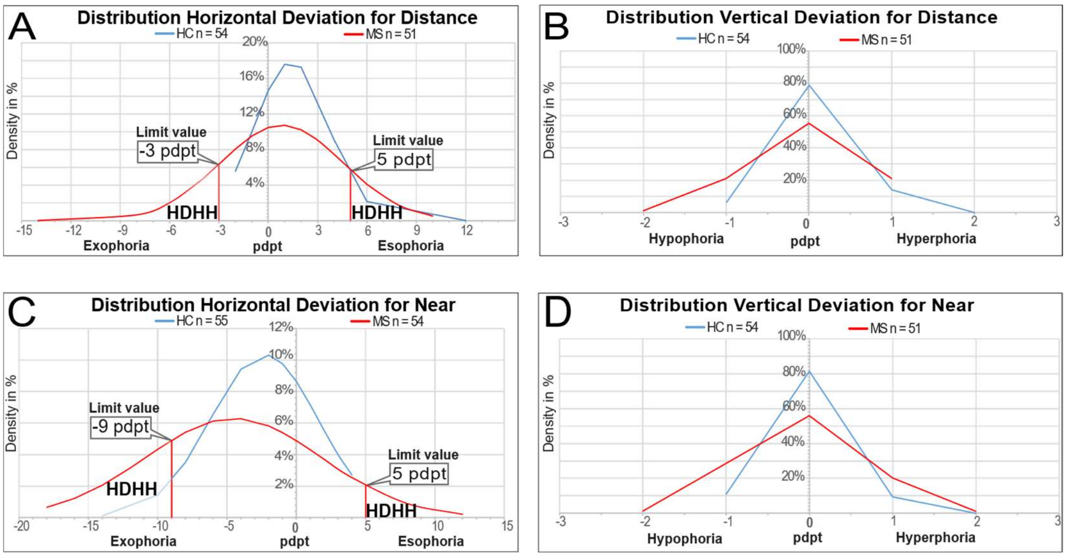

Supplementary Figure 5:

Distribution of high degrees of horizontal heterophoria (HDHH) of health controls (HCs; A) and multiple sclerosis (MS) patients (B) in relation to heterophoria inside of the defined limits, respectively for distance and near range.

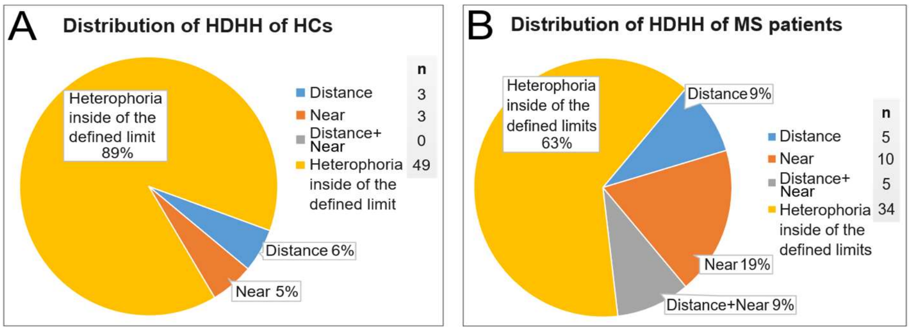

Supplement: Supplementary file 1 [file DataSheet1.pdf]
